# Differential Effects of Fingolimod and Natalizumab on Magnetic Resonance Imaging Measures in Relapsing–Remitting Multiple Sclerosis

**DOI:** 10.1007/s13311-021-01118-2

**Published:** 2021-09-24

**Authors:** S. Grahl, M. Bussas, B. Wiestler, P. Eichinger, C. Gaser, J. Kirschke, C. Zimmer, A. Berthele, B. Hemmer, M. Mühlau

**Affiliations:** 1 Department of Neurology, School of Medicine, Klinikum Rechts der Isar, Technical University of Munich, Ismaninger Str. 22, 81675 Munich, Germany; 2 TUM Neuroimaging, Technical University of Munich, Ismaninger Str. 22, 81675 Munich, Germany; 3 Department of Neuroradiology, School of Medicine, Klinikum Rechts der Isar, Technical University of Munich, Ismaninger Str. 22, 81675 Munich, Germany; 4 Department of Psychiatry and Department of Neurology, Jena University Hospital, Jena, Germany; 5 Munich Cluster for Systems Neurology (SyNergy), Feodor-Lynen-Str. 17, 81377 Munich, Germany

**Keywords:** Multiple sclerosis, MRI, Fingolimod, Natalizumab, Atrophy, White matter lesion

## Abstract

Fingolimod and natalizumab are approved disease-modifying drugs in relapsing–remitting multiple sclerosis (RRMS). The two drugs have different modes of action and may therefore influence different aspects of MS-related tissue damage. In this retrospective cohort study, we longitudinally compared patients treated with fingolimod and patients treated with natalizumab by measures based on structural magnetic resonance imaging (MRI). We included patients with RRMS given that two standardized MRI scans under the same drug were available with an interval of at least 6 months both from therapy start to baseline scan and from baseline scan to follow-up scan. After matching for age, baseline and follow-up scans from 93 patients (fingolimod, 48; natalizumab, 45) were investigated. Mean follow-up time was 1.9 years. We determined the number of new white matter lesions as well as thalamic, cortical, and whole-brain atrophy. After scaling for time of the interscan interval, measures were analyzed by group comparisons and, to account for demographic and clinical characteristics, by multiple regression models and a binary logistic regression model. Compared to natalizumab, fingolimod treatment went along with more new white matter lesions (median [interquartile range, IQR] 0.0 [0.0; 0.7] vs. 0.0 [0.0; 0.0] /year; *p* < 0.01) whereas whole-brain atrophy was lower (median [IQR] 0.2 [0.0; 0.5] vs. 0.5 [0.2; 1.0] %/year; *p* = 0.01). These significant differences were confirmed by multiple regression models and the binary logistic regression model. In conclusion, our observation is compatible with stronger neuroprotective properties of fingolimod compared to natalizumab.

**Supplementary Information:**

The online version contains supplementary material available at 10.1007/s13311-021-01118-2.

## Introduction

Multiple sclerosis (MS) is an autoimmune inflammatory disease of the central nervous system. In most instances, the early stage of MS is characterized by unpredictable episodes of neurological deficits (relapses) as a consequence of new white matter (WM) lesions resulting from acute inflammation. The early stage of relapsing–remitting MS (RRMS) is usually followed by a gradual accumulation of neurological deficits independent of demyelinating attacks, albeit at highly variable intervals. This later stage, secondary progressive MS (SPMS), is less well-understood with neurodegenerative processes coming more and more into play. Beyond demyelinating WM lesions, various pathological processes in virtually all compartments of the central nervous system have been assumed to contribute to neurodegeneration and consecutive brain atrophy [1, 2].

In the last decade, more than ten disease-modifying drugs (DMDs) have become available for clinical use. These DMDs have different modes of action [3] suggesting the possibility of divergent effects on different aspects of MS-related tissue damage and, hence, of divergent effects measurable with longitudinal MRI scans. Demonstration of the latter would not only deepen the understanding of DMDs but also contribute to the aim of individualized therapy in MS. Fingolimod and natalizumab are two DMDs approved for highly active RRMS. Natalizumab prevents lymphocytes from crossing the blood–brain barrier by blocking the interaction between lymphocytes’ VLA4 receptor and its endothelial ligand vascular cell adhesion molecule. In contrast, fingolimod is an antagonist of the S1P receptor 1. It is assumed to prevent T cells from leaving the secondary lymph organs which decreases the number of circulating lymphocytes [4]. In accordance with these anti-inflammatory modes of action, both drugs have been demonstrated in randomized placebo-controlled clinical trials to efficiently reduce acute inflammatory activity, namely the number of relapses and the number of new WM lesions [5–7]. Of note, neuroprotective properties, potentially influencing atrophy rates, have also been ascribed to fingolimod [8]. We are aware of only one prospective phase IV trial directly comparing fingolimod and natalizumab [9]. Because of enrolment-related early study termination of this multicenter study after 1 year, only secondary endpoints other than brain atrophy were reported. Natalizumab was superior to fingolimod with regard to reducing relapses and WM lesion accumulation. This difference was also reported in a meta-analysis gathering indirect evidence from randomized controlled trials and observational head-to-head trials [10]. Concerning brain atrophy, we are aware of only one longitudinal study comparing fingolimod and natalizumab [11].

Against this backdrop, we comparatively investigated the effects of natalizumab and fingolimod on longitudinal measures derived from structural brain MRI in subgroups of a monocentric observational cohort study.

## Methods

### Patients

**Fig. 1:**
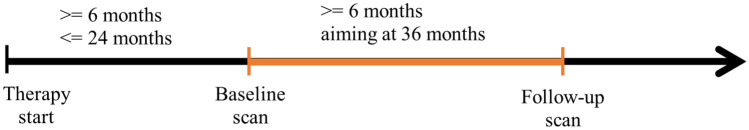
Scheme of the study design. MRI data analysis is illustrated by a time scale. The interval between therapy start was set to a minimum of 6 months and a maximum of 24 months. The observation period is marked in orange and was set to a minimum of 6 months and an optimum of 36 months when more than two scans were available

This retrospective analysis was part of the single-center cohort study on MS of the Technical University of Munich (TUM-MS), which was approved by the internal review board and performed in accordance with the Declaration of Helsinki. Patients had given written informed consent for the use of their clinical and paraclinical data for research purposes. We considered data of all patients included in TUM-MS. Inclusion criteria were a diagnosis of RRMS established by the treating physician, availability of at least two MRI scans under either fingolimod or natalizumab acquired at the same scanner with same standardized protocol. To exclude initial drug-related effects on brain volume (pseudoatrophy), the first (baseline) scan had to be at least 6 months after the initiation of the respective therapy. The maximum interval between treatment initiation and baseline scan was limited to 24 months. To ensure long and homogeneous observation periods, we also defined a minimal interval between scans of 6 months and, in cases of more than two scans, an optimal interval of 3 years. A schematic timeline is given in Fig. 1. To achieve comparable age ranges, we included only patients of an age within the intersecting age range of both groups. To evaluate the possibility of a selection bias due to treatment discontinuation before the end of month 6, we searched for patients, in whom one of the two treatments were initiated but discontinued. We included only patients in whom initiation of treatment was in the interval from 6 months before the earliest baseline scan (of all scans analyzed) to 6 months before the latest follow-up scan (of all scans analyzed).

### MRI Acquisition and Processing

Analyzed images were acquired at the same 3-T scanner (Achieva, Philips, Netherlands) according to our standardized protocol exclusively used between 2009 and 2017. Three-dimensional spoiled gradient echo T1-weighted (w) sequences were applied with the following parameters: voxel size = 1 mm isotropic, TR = 9 ms, TE = 4 ms. Furthermore, turbo-spin echo T2w fluid attenuated inversion recovery (FLAIR) images were acquired with the following parameters: voxel size = 1.0 × 1.0 × 1.5 mm; TR = 10,000 ms; TE = 140 ms; TI = 2750 ms. Primarily, all images were preprocessed and normalized with SPM12 and its toolboxes Computational Anatomy Toolbox (CAT, version 12.7, http://www.neuro.uni-jena.de/cat/index.html) and Lesion Segmentation Tool (LST, version 2.0.15, http://www.statistical-modeling.de/lst.html) with their default options resulting in T1w images which were bias-corrected and normalized to Montreal Neurological Institute space, and with their WM lesions filled with intensities of normal appearing WM as described earlier [12]. As implemented in CAT12, filled T1w images in native space were coregistered by the mean transformation of the longitudinal stream; then, thalamus volumes were calculated by a reverse mask approach based on a freely available atlas (Neuromorphometrics, Inc.). For comparison of baseline scans, thalamic volumes were scaled for total intracranial volumes (TIV) as output by the longitudinal stream of CAT12. Thalamus volumes were divided by the individual TIV and multiplied by the mean TIV of the whole cohort to keep values within an intuitive range. Global volumes of grey matter (GM) and WM at baseline were derived from CAT12 and divided by TIV to gain fractions of GM and WM. Baseline WM lesion volume was extracted from binarized WM lesion maps in native space with LST. Cortical thickness was calculated using the longitudinal stream of the surface toolbox in CAT12 [13]. All segmentations were visually checked. However, the longitudinal results on thalamic atrophy showed high variability. This let us repeat this analysis by the longitudinal streams of other software packages in the context of the review process. We used FSL (version 5.0.1, https://fsl.fmrib.ox.ac.uk/fsl) and FreeSurfer (version 6.0.0, http://surfer.nmr.mgh.harvard.edu). With these software packages, processing results were not satisfactory in few datasets that were excluded from analyses (fingolimod/natalizumab: FSL 1/0, FreeSurfer 1/2).

To assess brain atrophy, percentage brain volume change (PBVC), as implemented in the software package FSL SIENA (https://fsl.fmrib.ox.ac.uk/fsl/fslwiki/SIENA), was used. We accounted only for the central area of the brain (ranging from z-coordinates − 10 to + 60). This central slab method has been proven to produce similar statistical dispersion and correlations to clinical outcomes, compared to the whole-brain PBVC, but might be less affected by MR artifacts, partial volume, or motion effects [14]. SIENA calls a series of other FSL routines to prepare the MR images for PBVC estimation. To adapt all FSL steps to images from our scanner, we changed the fractional intensity threshold within BET (brain extraction tool, https://fsl.fmrib.ox.ac.uk/fsl/fslwiki/BET) to 0.06.

The number of new WM lesions was assessed based on FLAIR subtraction images (follow-up scan–baseline scan) as previously described [15]. In short, a custom-built script was used to calculate FLAIR subtraction images. First, FLAIR images of both MRI time points were rigidly coregistered using SPM12, with the follow-up FLAIR scan set as reference and the baseline FLAIR scan as source image. Second, both FLAIR images were brain extracted using FSL BET. Third, we scaled intensity of both FLAIR images by dividing them by their respective median intensity value. The subtraction image was calculated as a fourth step by subtracting the first from the second FLAIR image in RStudio (version 3.6.3, 2020). The number of new lesions was counted from these subtraction images manually and blinded for treatment group with a python-based tool [15].

### Statistical Analysis

To characterize both treatment groups, we first compared demographic and clinical data. Sex distribution was compared by Fisher’s exact test and the type of previous treatment by chi-square test (no treatment; first-line treatments: beta-interferons, glatiramer acetate, teriflunomide, dimethyl fumarate; second-line treatments: fingolimod, natalizumab). Otherwise, normally distributed variables (according to the Shapiro–Wilk test) were analyzed by two-sample *t*-tests and non-parametric variables by Wilcoxon tests. Second, we likewise compared MRI baseline and (longitudinal) outcome measures, between groups. We primarily focused on the most established paraclinical parameters in MS research, namely the number of new WM lesions, and brain atrophy (PBVC). Since, in early MS, brain atrophy develops primarily in GM [16–18], thalamic atrophy (change in thalamic volume) and cortical atrophy (change in cortical thickness) were analyzed subordinately to identify the GM compartment whose atrophy is reflected by PBVC. Atrophy measures were scaled so that more atrophy goes along with higher (positive) values. All MRI outcome parameters were also scaled for the time of the interscan interval to account for different observation periods. Apart from PBVC, atrophy measures (changes in thalamic volume and in cortical thickness) were normalized through division by the respective baseline value to account for differences in baseline values. Accordingly, longitudinal atrophy measures are given in percent per year (%/year) and the numbers of new WM lesions in lesions per year (/year). Third, significant comparisons of MRI outcome parameters between treatment groups were repeated by multiple linear regression models to control for potential confounders. MRI-based outcome parameters (as detailed above) served as response variables. In each model, age and those baseline parameters that differed significantly between groups served as covariates (if not accounted for by scaling). Finally, we setup a binary logistic regression model comprising all confounders, and significantly different MRI parameters as explanatory variables and treatment group as response variable.

For all statistical analyses, Rstudio version 3.6.3 (2020) was used and *p* values < 0.05 were considered statistically significant. Two-sided *p* values are given if not indicated otherwise. Normally distributed measures are given in mean ± standard deviation (SD) and non-normally distributed data in median and interquartile range (IQR).

## Results

### Characteristics of Patients

Analyzed were scans of 48 patients under fingolimod and 45 patients under natalizumab. At baseline, both groups did not differ in age (*p* = 0.14), sex distribution (*p* = 1.0), EDSS score (*p* = 0.1), time from treatment start (*p* = 0.4), and WM lesion volume (*p* = 0.2). Patients under fingolimod had significantly longer disease durations (6.9 ± 5.6 vs. 5.0 ± 4.1, *p* = 0.03) and significantly longer observation periods (time between baseline and follow-up scans, *p* < 0.01). Natalizumab patients had more relapses in the year before treatment start (*p* < 0.01), had been treated less frequently with another DMD beforehand (*p* = 0.01), and had lower global GM volumes (*p* < 0.02); during the observation period, relapses were rare in both groups (Table 1). In our database, we identified 17 patients in whom one of the two treatments were discontinued before the end of month 6 for different reasons (fingolimod: side effects, 8; disease activity, 4; pregnancy, 1. Natalizumab: side effects, 1; compliance, 1; change of patient’s preference in the light positivity for JC virus antibodies despite prior consent, 2).

**Table 1:** Demographic and clinical characteristics and baseline MRI measures

	Fingolimod	Natalizumab	*p* value
*N*	48	45	
Age in years	37.3 (7.8)	34.4 (8.6)	0.1
Female (%)	34 (70.8)	31 (68.9)	1.0
EDSS at baseline MRI			
Mean (SD)	1.6 (1.2)	1.9 (1.2)	
Median (IQR)	1.5 (1.0; 2.3)	2.0 (1.5; 2.5)	0.1
Disease duration in years			
Mean (SD)	6.9 (5.6)	5.0 (4.1)	
Median (IQR)	5.4 (3.3; 8.3)	4.2 (1.8; 7.1)	*0.03*
ARR in the year before treatment			
Mean (SD)	1.0 (1.1)	1.5 (1.0)	
Median (IQR)	1.0 (0.0; 1.3)	1.0 (1.0; 2.0)	< *0.01*
ARR during treatment			
Mean (SD)	0.05 (0.2)	0.09 (0.2)	
Median (IQR)	0.00 (0.0; 0.0)	0.00 (0.0; 0.0)	0.2
Previous treatment in %			
None/first line/second line	6/73/21	29/58/13	*0.01*
Time between treatment start and baseline scan in years			
Mean (SD)	0.9 (0.3)	0.9 (0.2)	
Median (IQR)	0.9 (0.7; 1.0)	0.9 (0.8; 1.0)	0.4
Interscan interval in years			
Mean (SD)	2.2 (1.3)	1.7 (0.8)	
Median (IQR)	2.0 (1.0; 3.0)	1.5 (1.0; 2.2)	*0.02*
WML volume at baseline scan in milliliters			
Mean (SD)	9.5 (10.9)	11.9 (11.1)	
Median (IQR)	5.2 (2.1; 11.9)	9.0 (3.9; 15.5)	0.2
Total intracranial volume, baseline, milliliters			
Mean (SD)	1504 (148.9)	1526 (136.4)	
Median (IQR)	1493 (1384; 1598)	1504 (1445; 1603)	0.4
Grey matter fraction at baseline			
Mean (SD)	0.43 (0.03)	0.42 (0.02)	*0.02*
Median (IQR)	0.43 (0.4; 0.4)	0.42 (0.4; 0.4)	
White matter fraction at baseline			
Mean (SD)	0.31 (0.02)	0.31 (0.02)	0.2
Median (IQR)	0.31 (0.3; 0.3)	0.3 (0.3; 0.3)	
Cortical thickness at baseline in millimeters			
Mean (SD)	2.7 (0.1)	2.6 (0.1)	0.3
Median (IQR)	2.6 (2.6; 2.7)	2.6 (2.5; 2.7)	
Thalamus volume at baseline in milliliters			
Mean (SD)	9.2 (1.8)	8.5 (1.6)	
Median (IQR)	9.5 (7.5; 10.6)	8.7 (7.6; 9.5)	*0.05*

Values are given in mean (standard deviation) and in median (interquartile range) *ARR*, annualized relapse rate; disease duration, time between first clinical event and baseline scan in years; *EDSS*, Expanded Disability Status Scale; *IQR*, interquartile range; *N*, number; *SD*, standard deviation; *TIV*, total intracranial volume; *WM*, white matter; *WML*, white matter lesion

### Comparison of Outcome Measures

**Fig. 2:**
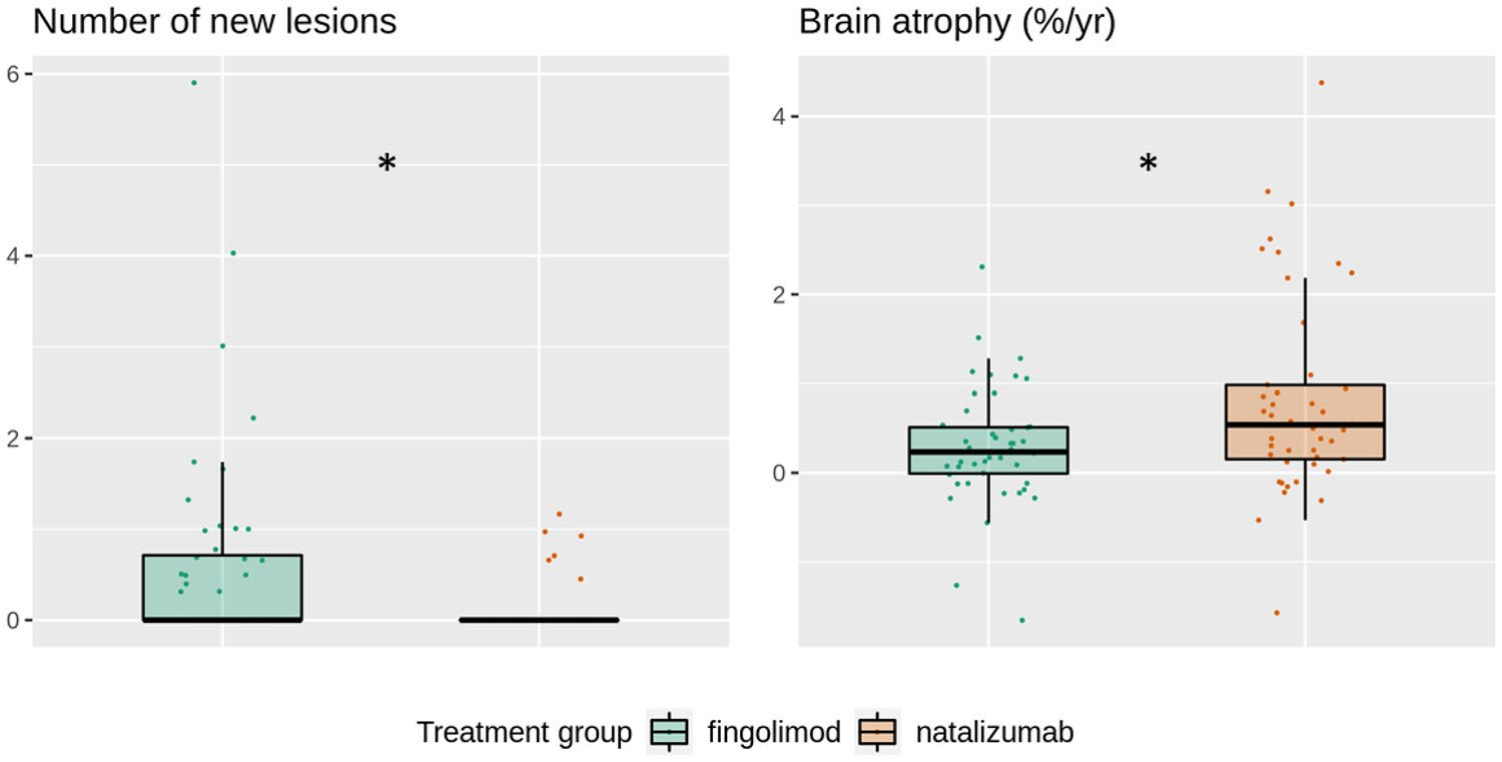
Comparison of longitudinal MRI measures between both treatment groups. Number of new lesions is annualized (division by interscan interval in years). Brain atrophy values (percentage brain volume changes) are normalized (division by interscan interval in years resulting in the unit %/year). Asterisks indicate significance at *p* < 0.05

**Fig. 3:**
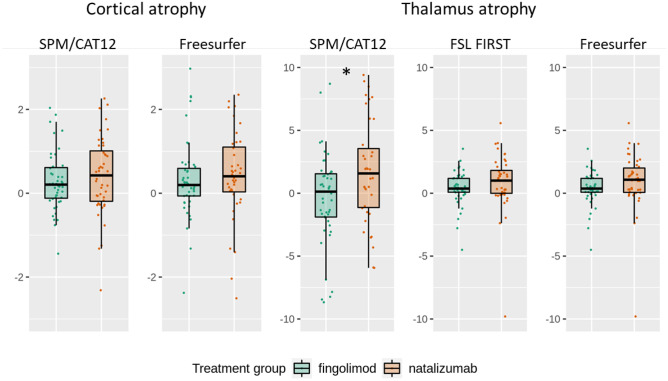
Comparison of longitudinal MRI measures of grey matter atrophy subordinate to brain atrophy. Cortical and thalamic atrophy values (annualized percentage changes of cortical thickness and thalamus volumes). Asterisks indicate significance at *p* < 0.05

Compared to natalizumab, fingolimod treatment went along with significantly higher numbers of new WM lesions whereas brain atrophy was significantly lower (Table 2, Fig. 2). The latter could not clearly be attributed to either cortical or subcortical (thalamic) atrophy. Cortical atrophy was also less pronounced in the fingolimod group, but this difference was not significant. The results on thalamic atrophy were not consistent. On the one hand, the results based on SPM/CAT12 suggested significantly lower atrophy under fingolimod than under natalizumab. On the other hand, atrophy rates seemed to range from almost + 10% to almost − 10%, which let us repeat the analyses with the software FSL and FreeSurfer in the context of the review process. Again, results indicated less thalamic atrophy under fingolimod than under natalizumab; however, significance was marginal when using the software FSL and FreeSurfer (Table 2, Fig. 3) with *p* values of 0.09 and 0.08, respectively (corresponding to one-sided *p* values of < 0.05).

**Table 2:** Direct group comparisons of outcome measures

	Fingolimod	Natalizumab	*p* value
*N*	48	45	
*N* new lesions/year			
Mean (SD)	0.6 (1.2)	0.1 (0.3)	
Median (IQR)	0.0 (0.0; 0.7)	0.0 (0.0; 0.0)	*1e-3*
Brain atrophy %/year			
Mean (SD)	0.3 (0.6)	0.8 (1.1)	
Median (IQR)	0.2 (0.0; 0.5)	0.5 (0.2; 1.0)	*0.01*
Measures of grey matter atrophy (subordinate to brain atrophy)			
Cortex atrophy %/year (CAT12)			
Mean (SD)	0.1 (1.1)	0.5 (1.2)	
Median (IQR)	0.2 (− 0.1; 0.6)	0.5 (− 0.2; 1.1)	0.21
Cortex atr. (FreeSurfer, *n* = 47/43)			
Mean (SD)	0.3 (0.9)	0.4 (1.4)	
Median (IQR)	0.2 (− 0.1; 0.6)	0.4 (0.0; 1.2)	0.22
Thalamus atrophy %/year (CAT12)			
Mean (SD)	− 1.4 (7.9)	3.4 (6.1)	
Median (IQR)	0.1 (− 2.0; 1.5)	1.9 (− 1.0; 7.5)	*1e-3*
Thalamus atr. (FreeSurfer, *n* = 47/43)			
Mean (SD)	0.7 (1.1)	1.1 (1.4)	
Median (IQR)	0.5 (0.1; 1.0)	0.8 (0.2; 1.7)	0.08
Thalamus atrophy (FSL, *n* = 47/45)			
Mean (SD)	0.4 (1.3)	1.1 (2.7)	
Median (IQR)	0.4 (0.1;1.2)	1.0 (0.0;2.1)	0.09

Values are given in mean (standard deviation, SD) and in median (interquartile range, IQR) */year*, change per year; *%/year*, atrophy in percent per year; *atr.*, atrophy

Regarding new WM lesions and brain atrophy, significant differences between treatment groups were confirmed by multiple linear regression models in which possible confounders were included (Table 3). These models revealed further associations with MRI outcome parameters. As expected, higher age went along with faster brain atrophy and less disease activity as indicated by fewer new WM lesions. Finally, new WM lesions and brain atrophy were significantly related to treatment group in a single binary logistic regression model (Table 3).

**Table 3:** Association of outcome measures and treatment group derived from multiple linear regression models and from a single binary logistic regression model

**Multiple regression models**			
Response variable	Explanatory variables	Standardized *ß*	*p* value
New lesions /year	Treatment group	−0.34	*2e-3*
	Age	−0.24	*0.02*
	Disease duration	−0.03	0.77
	Prior relapses	0.03	0.78
	Prior treatment*	0.02	0.84
Brain atrophy %/year	Treatment group	0.37	*6e-4*
	Age	0.22	*0.03*
	Disease duration	−0.15	0.17
	Prior relapses	−0.24	*0.02*
	Prior treatment*	0.05	0.64
**Binary logistic regression model**			
Response variable	Explanatory variables	Standardized *ß*	*p* value
Treatment group	Brain atrophy %/year	1.12	*0.002*
	New lesions /year	−1.7	*0.01*
	Age	−0.66	*0.03*
	Disease duration	0.04	0.9
	Prior relapses	0.67	*0.02*
	Prior treatment*	−0.53	0.09

Associations of outcome measures with treatment group (coded with 1 for fingolimod and 2 for natalizumab) and possible confounders are shown. Note that atrophy measures correspond to volume changes so that more atrophy goes along with higher (positive) values; in consequence, *ß* values of the variable treatment group favor natalizumab when negative and fingolimod when positive ^*^Prior treatment was coded by 0 (none), 1 (first line), and 2 (second line) */year*, per year; *%/year*, percent per year

## Discussion

In this retrospective cohort study, structural brain measures based on longitudinal MRI were compared between two well-established DMDs for the treatment of highly active RRMS, fingolimod and natalizumab. We investigated whether their different modes of action translate into differential effects at the level of brain structure focusing on brain atrophy as this would imply the possibility of neuroprotective properties and, hence, favorable long-term effects. In line with previous studies [9–11, 19], natalizumab showed stronger effects in limiting inflammation and demyelination as indicated by fewer new WM lesions. Our main finding is, however, that fingolimod showed stronger effects on slowing of brain atrophy. We will consider methodological issues of our study, relate our results to those reported in the literature, and acknowledge limitations of our study.

Regarding brain atrophy, we expected, if at all, small and subclinical effects only detectable by MRI-based measures. To increase statistical power, we opted for a possibly long cumulative observation interval and considered the availability of at least one pair of MRI scans sufficient. To maximize individual interscan intervals and to exclude a meaningful influence of pseudoatrophy, we chose a minimum interval between therapy initiation and baseline scan of 6 months. Several studies reported pseudoatrophy in the first year of treatment with natalizumab [20–23] and fingolimod [24]. The only study investigating the course of atrophy within the first year of natalizumab treatment observed accelerated atrophy primarily during the first 6 months predominantly in patients with inflammatory activity [25]. A similar observation was made for fingolimod [24]. Therefore, we believe that these data justify our choice of a minimal interval from therapy initiation and baseline scan of 6 months [25]. We did not have a hypothesis on different effects across brain regions and, therefore, focused on global measures. We chose three well-established measures representing the whole brain (PBVC) as well as deep (thalamus) and cortical (thickness) GM, since early MS-related atrophy is pronounced in brain GM [18, 26]. In addition to the patient selection, we accounted for imbalances in baseline characteristics by a three-step analysis. It comprised simple comparisons of MRI outcome measures as well as correction for significantly different baseline parameters through multiple linear regression models and through a single binary logistic model. Weighing potential confounders, we prioritized age in the selections step as it considerably influences the course of MS [27–29] and the speed of GM loss even in normal aging [30, 31]. To this end, we selected only patients of an age within the overlapping range of both treatment groups. For statistical analyses, we regarded the direct comparison of MR outcome parameters between groups valuable for three reasons. First, all outcome variables were scaled for the time of the observation interval; second, new WM lesions is a well-established outcome parameter; and third, all atrophy measures were additionally scaled for baseline values. Nevertheless, to account for potential confounders, we also performed multiple linear regression models including significantly different baseline parameters. Because of the huge effect sizes of age on brain GM volume, we also included age in the multiple linear regression models. Of note, statistical significance on group differences was higher in these multiple regression models suggesting that potential confounders explained variance of MR outcome parameters but did not drive group differences. Finally, we could demonstrate an independent association of new WM lesions and brain atrophy with the treatment group by a single binary logistic model. We therefore conclude that the effect of less whole-brain atrophy under fingolimod compared to natalizumab was robust in our cohort.

Our results of whole-brain atrophy (PBVC per year) are in the range of those reported in the literature. Our mean value of 0.3%/year under fingolimod complies with atrophy rates between 0.3 and 0.5%/year as reported in three large multicenter clinical trials [5, 7, 32]. Furthermore, there is evidence that mitigation of brain atrophy contributes to fingolimod’s effect on disability [33]. In contrast to fingolimod, atrophy rates under natalizumab vary largely across studies ranging from not detectable to more than 1%/year [20–23, 25, 34–37]. These studies were smaller apart from one large multicenter trial [22]. Pseudoatrophy [20–22, 34], most pronounced in the first 6 months [25], and the degree of inflammatory activity at the time of treatment initiation [21, 25] have been regarded responsible for high rates of brain atrophy. Yet several studies have reported atrophy rates under natalizumab larger than those under fingolimod also after the first year of treatment [20, 21, 35, 36] suggesting that brain atrophy rates under natalizumab may be larger than those under fingolimod beyond initial effects on inflammatory activity, i.e., beyond pseudoatrophy. Furthermore, the finding of another study that brain atrophy under natalizumab is independent of baseline inflammation and correlates with disability points in the same direction [34]. Our results are however in contrast to the study by Preziosa et al. [11], which is the only study, we are aware of, that directly compared atrophy measures under fingolimod with those under natalizumab [11]. In this prospective, non-randomized, open label, single-center trial, 25 patients under fingolimod were compared to 30 patients under natalizumab and no differences in brain atrophy rates (PBVC, total GM volume, deep GM volume change) were observed. We are currently unable to explain the discrepancy in results between the Preziosa study [11] and our study. Neither do we see a clear advantage of one study over the other. On the one hand, three scans per subject and well-balanced treatment groups are certainly an advantage of the study by Preziosa et al. [11]; on the other hand, the cumulative observation time, and hence statistical power, may have been higher in our study (182 years = 2.2 * 48 + 1.7 * 45 in our study vs. 55 years for the first and 55 years for the second year in the Preziosa study [11]).

We acknowledge limitations of our work beyond those inherent to retrospective cohort studies. We were not able to perfectly match groups and inclusion of parameters with significant differences between groups into statistical models may not have accounted for all aspects of group imbalance such as prior treatment and time of treatment before the baseline scan. To the cost of relatively large groups, MRI scans of only two time points were analyzed which leaves some uncertainty about the course of shifts in MRI parameters. The same applies to inflammatory activity at time of treatment initiation, in principle measurable through the administration of a Gadolinium-based contrast agent, which however is no longer routinely performed at our institution. We could not convincingly attribute differences in whole-brain atrophy (PBVC) to deep GM (thalamus) or cortical GM. In retrospect, this attempt seems overambitious, since it would have been necessary to reliably detect changes in cortical thickness far in the range of submillimeters and since segmentation of deep GM structures, including the thalamus, has been shown to be challenging in MS [38]. Finally, we could only compare patients who actually received the treatment throughout the defined interval. Some patients have discontinued treatment after initiation and before month 6, the time of the first scan of our analysis. This may have introduced a selection bias and contributed to differences in MRI-based measures not attributable to the different modes of actions of the two drugs.

We conclude that our results are in principle compatible with neuroprotective properties of fingolimod. However, these results are currently in conflict with results from another study [11] and need to be replicated in further datasets, ideally, containing data of more patients and longer observations periods. Most likely, such an analysis necessitates a multicenter design.

## Supplementary Information

Below is the link to the electronic supplementary material.

Supplementary file1 (PDF 511 KB)

Supplementary file2 (PDF 511 KB)

Supplementary file3 (PDF 511 KB)

Supplementary file4 (PDF 511 KB)

Supplementary file5 (PDF 511 KB)

Supplementary file6 (PDF 511 KB)

Supplementary file7 (PDF 511 KB)

Supplementary file8 (PDF 511 KB)

Supplementary file9 (PDF 1752 KB)

Supplementary file10 (PDF 1753 KB)

## References

[1] Thompson AJ, Banwell BL, Barkhof F (2018). Diagnosis of multiple sclerosis: 2017 revisions of the McDonald criteria. Lancet Neurol..

[2] Reich DS, Lucchinetti CF, Calabresi PA (2018). Multiple Sclerosis. N Engl J Med..

[3] McGinley MP, Goldschmidt CH, Rae-Grant AD (2021). Diagnosis and Treatment of Multiple Sclerosis: A Review. JAMA..

[4] Tintore M, Vidal-Jordana A, Sastre-Garriga J (2019). Treatment of multiple sclerosis - success from bench to bedside. Nat Rev Neurol..

[5] Kappos L, Radue EW, O’Connor P (2010). A placebo-controlled trial of oral fingolimod in relapsing multiple sclerosis. N Engl J Med..

[6] Polman CH, O’Connor PW, Havrdova E (2006). A randomized, placebo-controlled trial of natalizumab for relapsing multiple sclerosis. N Engl J Med..

[7] Calabresi PA, Radue EW, Goodin D (2014). Safety and efficacy of fingolimod in patients with relapsing-remitting multiple sclerosis (FREEDOMS II): a double-blind, randomised, placebo-controlled, phase 3 trial. Lancet Neurol..

[8] Chun J, Giovannoni G, Hunter SF (2021). Sphingosine 1-phosphate Receptor Modulator Therapy for Multiple Sclerosis: Differential Downstream Receptor Signalling and Clinical Profile Effects. Drugs..

[9] Butzkueven H, Licata S, Jeffery D, et al. Natalizumab versus fingolimod for patients with active relapsing-remitting multiple sclerosis: results from REVEAL, a prospective, randomised head-to-head study. BMJ Open. 2020;10:e038861. 10.1136/bmjopen-2020-038861 PMC7577060 33082194

[10] Tsivgoulis G, Katsanos AH, Mavridis D, et al. The Efficacy of Natalizumab versus Fingolimod for Patients with Relapsing-Remitting Multiple Sclerosis: A Systematic Review, Indirect Evidence from Randomized Placebo-Controlled Trials and Meta-Analysis of Observational Head-to-Head Trials. PLoS One. 2016;11:e0163296. 10.1371/journal.pone.0163296 PMC5042498 27684943

[11] Preziosa P, Rocca MA, Riccitelli GC (2020). Effects of Natalizumab and Fingolimod on Clinical, Cognitive, and Magnetic Resonance Imaging Measures in Multiple Sclerosis. Neurotherapeutics..

[12] Biberacher V, Schmidt P, Keshavan A (2016). Intra- and interscanner variability of magnetic resonance imaging based volumetry in multiple sclerosis. NeuroImage..

[13] Dahnke R, Yotter RA, Gaser C (2013). Cortical thickness and central surface estimation. NeuroImage..

[14] Ruberte E, Sinnecker T, Amann M (2018). Central Slab versus Whole Brain to Measure Brain Atrophy in Multiple Sclerosis. Eur Neurol..

[15] Eichinger P, Wiestler H, Zhang H (2017). A novel imaging technique for better detecting new lesions in multiple sclerosis. Journal of neurology..

[16] Audoin B, Zaaraoui W, Reuter F (2010). Atrophy mainly affects the limbic system and the deep grey matter at the first stage of multiple sclerosis. Journal of neurology, neurosurgery, and psychiatry..

[17] Chard DT, Griffin CM, Rashid W (2004). Progressive grey matter atrophy in clinically early relapsing-remitting multiple sclerosis. Mult Scler..

[18] Raz E, Cercignani M, Sbardella E (2010). Gray- and white-matter changes 1 year after first clinical episode of multiple sclerosis: MR imaging. Radiology..

[19] Preziosa P, Rocca MA, Pagani E (2020). Two-year regional grey and white matter volume changes with natalizumab and fingolimod. Journal of neurology, neurosurgery, and psychiatry..

[20] Vidal-Jordana A, Sastre-Garriga J, Perez-Miralles F (2013). Early brain pseudoatrophy while on natalizumab therapy is due to white matter volume changes. Mult Scler..

[21] Sastre-Garriga J, Tur C, Pareto D (2015). Brain atrophy in natalizumab-treated patients: A 3-year follow-up. Mult Scler..

[22] Miller DH, Soon D, Fernando KT (2007). MRI outcomes in a placebo-controlled trial of natalizumab in relapsing MS. Neurology..

[23] Eisele P, Szabo K, Ebert A, Platten M, Gass A. Brain Atrophy in Natalizumab-treated Patients with Multiple Sclerosis: A 5-year Retrospective Study. J Neuroimaging. 2018. 10.1111/jon.12586 30485572

[24] De Stefano N, Silva DG, Barnett MH. Effect of Fingolimod on Brain Volume Loss in Patients with Multiple Sclerosis. CNS Drugs. 2017;31:289-305. 10.1007/s40263-017-0415-2 PMC5374177 28247239

[25] Magraner M, Coret F, Casanova B (2012). The relationship between inflammatory activity and brain atrophy in natalizumab treated patients. Eur J Radiol..

[26] Dalton CM, Chard DT, Davies GR (2004). Early development of multiple sclerosis is associated with progressive grey matter atrophy in patients presenting with clinically isolated syndromes. Brain..

[27] Dahlke F, Arnold DL, Aarden P, et al. Characterisation of MS phenotypes across the age span using a novel data set integrating 34 clinical trials (NO.MS cohort): Age is a key contributor to presentation. Mult Scler. 2021;0:1352458520988637. 10.1177/1352458520988637 PMC8564259 33507835

[28] Schwehr NA, Kuntz KM, Butler M (2020). Age-related decreases in relapses among adults with relapsing-onset multiple sclerosis. Multiple Sclerosis Journal..

[29] Confavreux C, Vukusic S (2006). Natural history of multiple sclerosis: a unifying concept. Brain..

[30] Vinke EJ, Huizinga W, Bergtholdt M (2019). Normative brain volumetry derived from different reference populations: impact on single-subject diagnostic assessment in dementia. Neurobiology of Aging..

[31] Frangou S, Modabbernia A, Williams SCR, et al. Cortical thickness across the lifespan: Data from 17,075 healthy individuals aged 3–90 years. Human Brain Mapping. 2021. 10.1002/hbm.25364 PMC8675431 33595143

[32] Cohen JA, Barkhof F, Comi G (2010). Oral fingolimod or intramuscular interferon for relapsing multiple sclerosis. N Engl J Med..

[33] Sormani MP, De Stefano N, Francis G (2015). Fingolimod effect on brain volume loss independently contributes to its effect on disability. Mult Scler..

[34] Ciampi E, Pareto D, Sastre-Garriga J (2017). Grey matter atrophy is associated with disability increase in natalizumab-treated patients. Mult Scler..

[35] Koskimaki F, Bernard J, Yong J, et al. Gray matter atrophy in multiple sclerosis despite clinical and lesion stability during natalizumab treatment. PLoS One. 2018;13:e0209326. 10.1371/journal.pone.0209326 PMC6303064 30576361

[36] Zivadinov R, Hojnacki D, Bergsland N, et al. Effect of natalizumab on brain atrophy and disability progression in multiple sclerosis patients over 5 years. Eur J Neurol. 2016. 10.1111/ene.12992 26998905

[37] Alvarez E, Nair KV, Hoyt BD, et al. Brain atrophy rates in patients with multiple sclerosis on long term natalizumab resembles healthy controls. Mult Scler Relat Disord. 2021;55:103170. 10.1016/j.msard.2021.103170 34364034

[38] de Sitter A, Verhoeven T, Burggraaff J (2020). Reduced accuracy of MRI deep grey matter segmentation in multiple sclerosis: an evaluation of four automated methods against manual reference segmentations in a multi-center cohort. Journal of Neurology..

